# Supplementary material for: PhenoComb: a discovery tool to assess complex phenotypes in high-dimensional single-cell datasets
Source: Bioinform Adv. 2022 Aug 3;2(1):vbac052. doi: 10.1093/bioadv/vbac052 (PMC9710698; doi:10.1093/bioadv/vbac052)
Supplement: vbac052_Supplementary_Data [file vbac052_supplementary_data.zip › Gating_Supplemental_Figure.pdf]

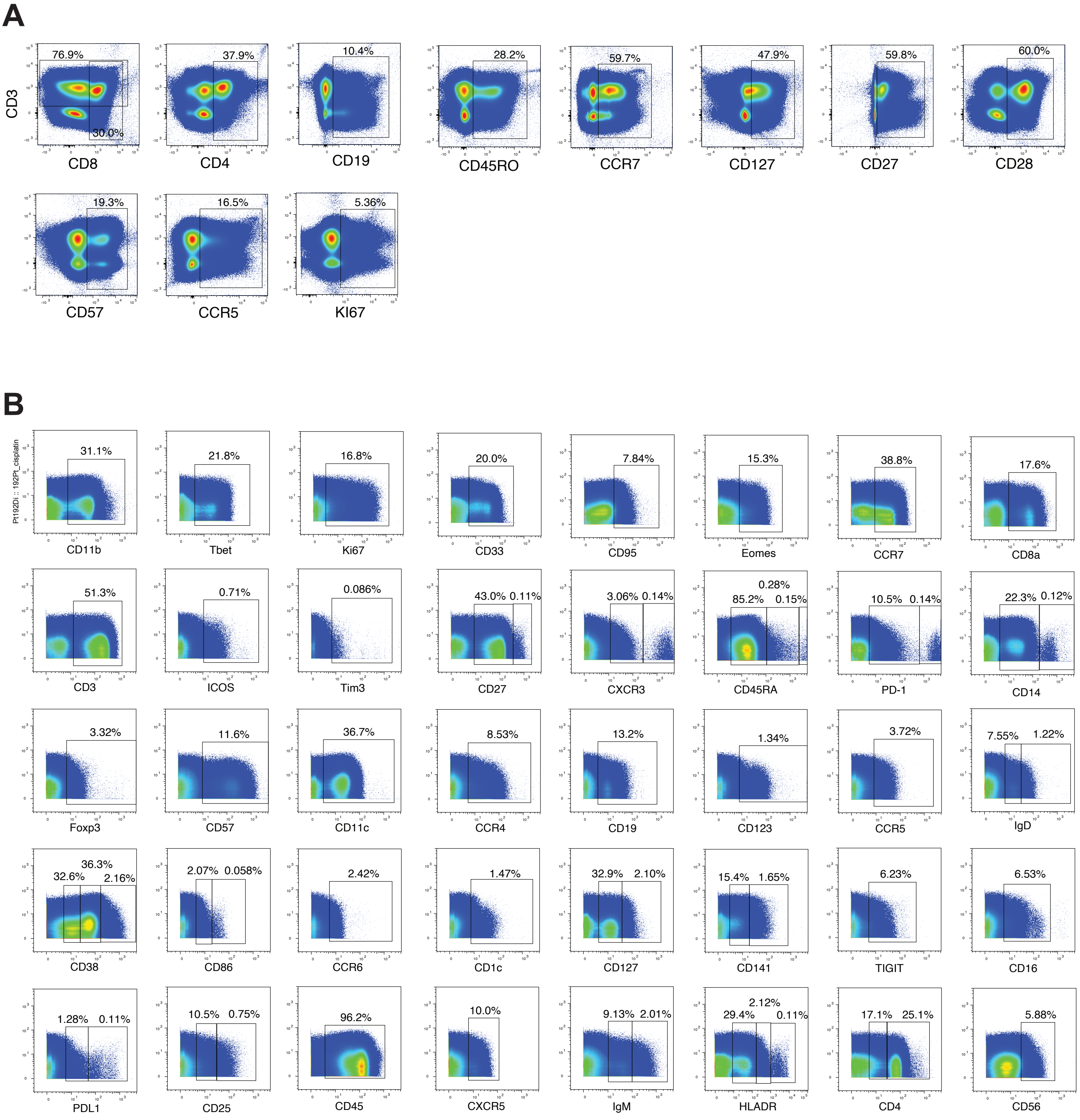

Supplemental Figure 1: **Gating Strategies.** Gating strategies for the **(A)** HIV dataset and for **(B)** COVIDome dataset. The rectangle's limits on the x axis are used as thresholds for obtaining discrete marker states.
