# Supplementary material for: PhenoComb: a discovery tool to assess complex phenotypes in high-dimensional single-cell datasets
Source: Bioinform Adv. 2022 Aug 3;2(1):vbac052. doi: 10.1093/bioadv/vbac052 (PMC9710698; doi:10.1093/bioadv/vbac052)
Supplement: vbac052_Supplementary_Data [file vbac052_supplementary_data.zip › COVIDdome_Marker_Table.pdf]

Supplemental Table 1

Lineages from COVIDome dataset.

	CD45+CD3+	CD45+CD19+	CD45+CD56+	CD45+CD14+	CD45+CD3-CD19-CD56-CD14-
CD45					
CD57	X	X	X	X	X
CD11c		X		X	X
CD16		X	X	X	X
CD196 CCR6	X	X	X	X	X
CD19					
CD123	X	X	X	X	X
CCR5	X	X	X	X	X
IgD		X			X
CD1c		X		X	X
CD38	X	X	X	X	X
CD127	X				
CD86		X		X	X
ICOS	X	X			
CD141				X	X
TGIT	X		X		
Tim3	X				
CD27	X				
CXCR3	X				
CD45RA	X	X			
PD-1	X	X			
PDL1	X	X	X	X	X
CD14					
Tbet	X	X	X		
Ki67	X	X	X	X	X
CD33				X	X
CD95	X	X			
Foxp3	X	X			
Eomes	X		X		
CCR7	X				
CD8a	X		X		
CD25	X	X			
CD3			X		
CXCR5	X	X	X	X	X
IgM		X			X
HLA-DR	X	X	X	X	X
CD4	X		X		
CCR4	X				
CD56					
CD11b				X	X
**Total markers**	**26**	**22**	**16**	**16**	**18**

*X = included in analysis
